# An evaluation of a community-based vision care programme for the elderly

**DOI:** 10.1186/s12877-022-03399-4

**Published:** 2022-08-27

**Authors:** She Chiu Yang, Tsz Kin Law, Yan Lok Lucas Leung, Yim Ying Tam, Rita Sum, Jinxiao Lian, Maurice Yap

**Affiliations:** School of Optometry, The Hong Kong Polytechnic University, Hung Hom, Kowloon, Hong Kong

**Keywords:** Vision care, Visual impairment, Elderly population, Health services research, Cost-effectiveness

## Abstract

**Background:**

This study evaluated the real-world effectiveness and potential cost-effectiveness of a community-based vision care programme for the elderly population aged 60 years or above.

**Methods:**

Data from a total of 8899 subjects participating in a community-based comprehensive vision care programme from 2015 to 2019 were analysed to evaluate the effectiveness of the programme in terms of the prevalence of distance visual impairment (VI), the change in the prevalence of distance VI after refractive error correction, and the types of ocular disorders suspected. Distance VI was defined as a) visual acuity (VA) worse than 6/18 in any eye (worse eye) and b) VA worse than 6/18 in the better eye. The cost-effectiveness from the funder’s perspective was also estimated in terms of cost per distance VI avoided.

**Results:**

Based on the presenting vision of the worse eye, the prevalence of distance VI was 39.1% (3482/8899, 95% CI: 38.1%-40.1%) and reduced to 13.8% (1227/8899, 95% CI: 13.1%-14.5%) based on best-corrected VA. Referenced to the presenting vision of the better eye, the prevalence of distance VI was 17.3% (1539/8899, 95% CI: 16.5%-18.1%) and decreased to 4.2% (373/8899, 95% CI: 3.8%-4.6%) with best optical correction. Uncorrected refractive error was the major cause of presenting distance VI. From the funder’s perspective, the cost per distance VI case prevented was HK$1921 based on VA in the worse eye and HK$3715 based on the better eye.

**Conclusion:**

This community-based programme identified distance VI in the best eye of 17 out of every 100 subjects. With appropriate new or updated distance optical corrections, distance VI was reduced to about 4 in 100 subjects. Visual impairment in the elderly is common even in a relatively affluent city. A model of care which could minimise avoidable distance VI would bring benefits at individual and societal levels.

## Background

According to the World Health Organization (WHO), the proportion of the global population over 60 years old will increase from 12 to 22% between 2015 and 2050 [1]. Ageing is associated with general functional loss and impairment, including vision [2]. Age-related vision problems and disorders, such as uncorrected refractive error, cataracts, age-related macular degeneration, and diabetic retinopathy, are thus expected to become more common. Poor vision affects daily living activities and quality of life and often results in negative consequences such as poor mental health, falls, and increased dependency [3, 4]. More people are expected to experience visual impairment in a society with an ageing population. There is urgency for healthcare and social systems to prepare for this demographic shift in the future.

From 2019 to 2050, the population of adults aged 60 years or above in Hong Kong (HK) is estimated to increase from 25 to 40% [5]. A 2002 population-based study of 3441 elders aged 60 years or above in the Shatin district, a suburban area of HK, found that 41.3% presented with visual acuity (VA) less than 6/18 in at least one eye [6]. This percentage of visual impairment (VI) dropped to 34.5% when a pinhole was used. In addition, 19.5% of subjects presented with VA less than 6/18 in the better eye. The main causes of VI were uncorrected refractive error, cataract and macular degeneration. Over the ensuing 20 years, public healthcare sector spending has been increasing by around 7% per annum which has ensured modern healthcare services are available to all [7]. All these may contribute to improved access to vision care and prevention of VI. A more recent cross-sectional study conducted between 2016 and 2018 found that the prevalence of VI (i.e. VA worse than 20/60 in the better eye) among HK elderly aged 60 years or above, who were recruited from the FAMILY cohort project, was 9.5% (63/661) and 2.0% (13/661) for presenting vision and best-corrected VA respectively [8]. Similarly, uncorrected refractive error and cataracts were the major causes of VI. However, this study has several features which make a direct comparison with the Shatin study problematic. First, the subjects recruited were aged from between 18 to 90 years. With lower participation from older subjects, this study is likely to underestimate the prevalence rate of VI in the general population. Second, the subjects were recruited from a family cohort with some coming from the same family and this raises the potential of the family clustering effect [8].

The WHO has called for global action to support healthy ageing by formulating evidence-based policies that strengthen the abilities of older persons and align health systems with the needs of older populations [1]. Older people who maintain good vision are more likely to maintain good functioning in activities of daily living, good social function, and independence. They are also more likely to avoid co-morbidities related to poor visual function such as falls and depression [9]. A community-based programme, namely the “Kwai Tsing Signature Project Scheme” (KT programme), was established in 2015 in the Kwai Tsing district in HK with the purpose to provide comprehensive vision care to the elderly residents of the Kwai Tsing district. This provided an opportunity to evaluate the real-world evidence from a community health service in terms of the effectiveness and potential cost-effectiveness of addressing the needs for vision care for an ageing population.

## Methods

### Community-based programme

This KT programme was launched by the Kwai Tsing District Council, in collaboration with the Kwai Tsing Safety Community and Healthy City Association, and the School of Optometry of The Hong Kong Polytechnic University (PolyU). This programme was publicized over a wide area by the District Council through various channels, such as advertisements and public events. Under the scheme, Kwai Tsing residents aged 50 years or above could apply for a one-off comprehensive vision examination at the Integrative Community Health Centre (ICHC) in Lai King with a co-payment of HK$15 (HK$10 initially during 2015–2017, 1US$ = 7.8HK$) and purchase a pair of single-vision spectacles with a co-payment of HK$75 if indicated (the co-payment was HK$50 initially during 2015–2017). The District Council, with funding from the Home Affairs Bureau of the Hong Kong SAR government, subsidised HK$250 for each examination and HK$550 for a pair of prescription spectacles (the subsidy for spectacles was HK$500 initially during 2015–2017). Those residents who were under the Comprehensive Social Security Assistance (CSSA) Scheme or recipients of fringe benefits for civil servants and their relatives were not eligible for this KT programme. The comprehensive vision examination was conducted by optometrists in the ICHC of PolyU following the routine examination protocol, including history taking, VA measurement and preliminary testing, objective and subjective refraction, external and internal ocular health assessment and additional tests if indicated.

### Subjects and data extraction

All subjects who have participated in the KT programme, aged 60 years or above, and with complete examination records were eligible for this study. This study focused on the analysis of the distance visual acuity as distance vision is the usual parameter used in vision standards. The examination rooms were equipped with the Thomson Test Chart (Thomson Software Solutions, UK) which was displayed on a monitor and with the aid of a mirror, calibrated to be viewed at an optical distance of 6 m. In a typical measurement of presenting vision, the subject covered one eye with an occluder and read the letters of the chart until the smallest line they could read. The measurement was repeated for the other eye with a different chart. The results were recorded in Snellen notation.

Subject data were extracted from the electronic record system and included demographic data, entrance distance VA, best-corrected distance VA (BCVA), and the types of suspected ocular disorders. Two broad categories were adopted, namely, VA based on the a) worse eye or b) better eye. For each category, 4 levels of VI were defined (Table 1) [10]. Distance VI was defined as VA less than 6/18, as used by Michon et al. [6]. Suspected ocular disorders were classified into 3 categories: uncorrected refractive error, anterior disorders (e.g. cataract and corneal scars) and posterior disorders (e.g. macular hole, age-related macular degeneration and epiretinal membrane).

**Table 1:** Definition of distance visual impairment

Level of distance visual impairment (VI)	Visual acuity
None	6/18 or better
Moderate	< 6/18- 6/60
Severe	< 6/60- 6/120
Blindness	worse than 6/120

### Statistical analysis

Descriptive analyses were used to summarise the prevalence of VI with a 95% confidence interval (CI), the changes in the levels of VI after refractive error correction, and the suspected ocular disorders. The difference in the prevalence of VI between males/females and age groups was analysed using the Chi-square test. The SPSS statistical software version 26.0 was used for analyses.

### Estimating the cost and cost-effectiveness of the subsidised programme

This part of the analysis estimated the direct cost of the subsidy from such a programme from the funder’s perspective and the cost per distance VI avoided as the immediate effect of receiving the comprehensive vision examination. From the funder’s perspective, the direct subsidised cost of the examination and spectacles for correcting refractive errors was considered. The co-payment from subjects for the examination and the spectacles were also included, but as revenue to the funder. This analysis represents the short-term cost-effectiveness of the programme by considering the immediate benefit from the programme.

## Results

From 2015 to 2019, a total of 9149 adults participated in the KT programme. Two hundred and fifty subjects were excluded due to not meeting the aged criterion or incomplete data set, leaving 8899 subjects eligible to be included as subjects for this study. Of those eligible to be subjects, 61.9% (5507/8899) were female participants and 46.7% (4159/8899) were 70 years or above (Table 2).

**Table 2:** Demographic characteristics of the subject group by sex and age (*N* = 8899)

Parameters	n (%)
**Sex**	
Male	3392 (38.1)
Female	5507 (61.9)
**Age**	
60–64	1813 (20.4)
65–69	2927 (32.9)
70–74	2055 (23.1)
75 or above	2104 (23.6)

### Prevalence of distance visual impairment

#### a) Based on the worse eye

The overall prevalence of distance VI was 39.1% (3482/8899, 95% CI: 38.1%-40.1%) based on presenting VA of the worse eye and 13.8% (1227/8899, 95% CI: 13.1%-14.5%) based on BCVA respectively (Table 3). Optical correction reduced the prevalence of moderate distance VI from 33.0% (2937/8899) to 10.4% (925/8899), severe distance VI from 3.7% (329/8899) to 1.4% (125/8899), and blindness from 2.4% (216/8899) to 2.0% (177/8899).

**Table 3:** Prevalence of distance visual impairment based on presenting VA and BCVA

Level of distance visual impairment (VI)	Prevalence of distance visual impairment, n (%), *N* = 8899			
	***VA less than 6/18 in the worse eye***		***VA less than 6/18 in the better eye***	
	Based on presenting VA	Based on BCVA	Based on presenting VA	Based on BCVA
None (≤ 6/18)	5417 (60.9)	7672 (86.2)	7360 (82.7)	8526 (95.8)
Moderate (> 6/18 and ≤ 6/60)	2937 (33.0)	925 (10.4)	1480 (16.6)	359 (4.0)
Severe (> 6/60 and ≤ 6/120)	329 (3.7)	125 (1.4)	49 (0.6)	11 (0.1)
Blindness (> 6/120)	216 (2.4)	177 (2.0)	10 (0.1)	3 (0.03)
Sub-total of visually impaired	3482 (39.1)	1227 (13.8)	1539 (17.3)	373 (4.2)

#### b) Based on the better eye

The overall prevalence of VI was reduced from 17.3% (1539/8899, 95% CI: 16.5%-18.1%) based on presenting VA of the better eye to 4.2% (373/8899, 95% CI: 3.8%-4.6%) with the use of an optical correction (Table 3). Optical correction reduced the prevalence of moderate distance VI from 16.6% (1480/8899) to 4.0% (359/8899), severe distance VI from 0.6% (49/8899) to 0.1% (11/8899), and blindness from 0.1% (10/8899) to 0.03% (3/8899).

### Comparison by sex and age group

There was more distance VI in older age groups. Based on the worse eye, the prevalence of VI in the “60–64” group was 22.5% (407/1813) and for the “75 or above” group, it was 62.1% (1306/2104) (Table 4). Based on the better eye, the prevalence of VI increased from 8.4% (152/1813) to 32.9% (693/2104) from the increased age groups.

**Table 4:** Prevalence of distance visual impairment based on presenting VA by sex and age group

Parameters	Have visual impairment, n (%)	No visual impairment, n (%)	Total	*P*-value*
	**Visual impairment based on the worse eye (*N* = 8899)**			
Sex				0.041
Male	1373 (40.5)	2019 (59.5)	3392 (100.0)	
Female	2109 (38.3)	3398 (61.7)	5507 (100.0)	
Age				< 0.001
60–64	407 (22.4)	1406 (77.6)	1813 (100.0)	
65–69	920 (31.4)	2007 (68.6)	2927 (100.0)	
70–74	849 (41.3)	1206 (58.7)	2055 (100.0)	
75 or above	1306 (62.1)	798 (37.9)	2104 (100.0)	
	**Visual impairment based on the better eye (*N* = 8899)**			
Sex				0.588
Male	596 (17.6)	2796 (82.4)	3392 (100.0)	
Female	943 (17.1)	4564 (82.9)	5507 (100.0)	
Age				< 0.001
60–64	152 (8.4)	1661 (91.6)	1813 (100.0)	
65–69	334 (11.4)	2593 (88.6)	2927 (100.0)	
70–74	360 (17.5)	1695 (82.5)	2055 (100.0)	
75 or above	693 (32.9)	1411 (67.1)	2104 (100.0)	

^*^Chi-square test

Among subjects with distance VI based on the worse eye, the proportion of correctable vision from any VI to no VI was significantly higher in the younger age group than in the older groups, reduced from 79.6% (324/407) in the 60–64 aged group to 50.0% (653/1306) in the 75 or above aged group (*P*-value < 0.001, Table 5). When the distance VI was defined based on the better eye, 92.1% (140/152) VI can be correctable in the aged group 60–64 and 64.1% (444/693) in the aged group 75 or above.

**Table 5:** Proportion of correctable distance visual impairment by sex and age group

**Parameters**	**Correctable visual impairment, n (%)**	**Uncorrectable visual impairment, n (%)**	**Total**	*P*-value*
	**Distance visual impairment based on the worse eye (*n* = 3482)**			
Sex				0.020
Male	857 (62.4)	516 (37.6)	1373 (100.0)	
Female	1398 (66.3)	711 (33.7)	2109 (100.0)	
Age				< 0.001
60–64	324 (79.6)	83 (20.4)	407 (100.0)	
65–69	702 (76.3)	218 (23.7)	920 (100.0)	
70–74	576 (67.8)	273 (32.2)	849 (100.0)	
75 or above	653 (50.0)	653 (50.0)	1306 (100.0)	
	**Distance visual impairment based on the better eye (*n* = 1539)**			
Sex				0.755
Male	449 (75.3)	147 (24.7)	596 (100.0)	
Female	717 (76.0)	226 (24.0)	943 (100.0)	
Age				< 0.001
60–64	140 (92.1)	12 (7.9)	152 (100.0)	
65–69	289 (86.5)	45 (13.5)	334 (100.0)	
70–74	293 (81.4)	67 (18.6)	360 (100.0)	
75 or above	444 (64.1)	249 (35.9)	693 (100.0)	

^*^Chi-square test

### Suspected ocular disorders among uncorrectable distance visual impairment

Suspected ocular disorders were classified into anterior and posterior types. Among 3482 subjects with distance VI based on worse eye, 2255 had a correctable refractive error. The remaining were still visually impaired in at least one eye after refractive error correction, including 612 subjects with anterior disorders only, 235 with posterior disorders only, 146 with both anterior and posterior disorders and 234 subjects with unknown causes (Table 6).

**Table 6:** Suspected ocular disorders for those who have impairment based on the worse eye (*n* = 3482)

Suspected ocular disorders	n (%)
Uncorrected refractive error	2255 (64.8)
Anterior disorders only	612 (17.6)
Posterior disorders only	235 (6.7)
Both (anterior + posterior) disorders	146 (4.2)
Unknown causes	234 (6.7)

### Cost-effectiveness of the subsidised programme

From the funder’s perspective, the total cost of subsidising the vision examination was HK$2,091,265 ((HK$250-HK$15)*8899) and the total cost of subsidising the glasses was HK$2,240,323 ((HK$550-HK$75)*8899*53%) (around 53% required new prescription glasses), giving a total of HK$4,331,588. After refractive error correction (Table 3), a total number of 2255 subjects improved from any distance VI to no VI using a) VA based on the worse eye and a total of 1166 subjects using b) VA based on the better eye. Therefore, the cost per distance VI avoided from this community-based programme was estimated to be HK$1921 (US$246) based on the worse eye and HK$3715 (US$476) based on the better eye from the funder’s perspective (Table 7).

**Table 7:** Cost-effectiveness of the community-based vision care programme

	**Value**
**Estimation of the cost (HK$)**	
Total number of subjects	8899
Subsidised cost per comprehensive vision examination	250
Co-payment by subject per comprehensive vision examination	-15
***Sub-total cost of subsidising comprehensive vision examination for all subjects***	2,091,265
Subsidised cost per pair of glasses	550
Co-payment by subject per pair of glasses	-75
Proportion requiring new glasses	53%
***Sub-total cost of subsidising glasses of all subjects***	2,240,323
**Total cost (A)**	**4,331,588**
**Effectiveness**	
Total number of subjects improved from any distance VI to no VI (worse eye) **(B)**	2255
Total number of subjects improved from any distance VI to no VI (better eye) **(C)**	1166
**Cost-effectiveness ratio (HK$)**	
Cost per distance VI avoided (worse eye) (A/B)	1921
Cost per distance VI avoided (better eye) (A/C)	3715

*VI* Visual impairment

## Discussion

This study evaluated the effectiveness and potential cost-effectiveness of a community-based programme to detect and address the vision problems of an elderly population resident in one district in Hong Kong. The results provide real-world evidence which could inform service planning for vision care for the wider elderly population.

This government-funded programme was initiated by a district council and was widely publicized in the relevant district. The participating elders represent those who would normally respond to public health initiatives in their community. This programme required a very low co-payment for the examination and a pair of glasses, so the financial barrier was minimal. Although the subjects might not be representative of the whole elderly population in HK, we are confident that they represented the more connected and health-conscious group. From this programme, a relatively high prevalence of distance VI was found. About 17 out of every 100 elders had distance VI (based on the better eye). With refractive error correction, distance VI improved to no VI in 76% of these cases. This significant drop suggests that a majority of the elders with distance VI benefited from this type of community-based programme and particularly so for the groups with moderate to severe VI and the younger aged groups. Results from another study of people aged 60 years and above in another part of HK indicated a 78.9% reduction in VI after optical correction [8].

The prevalence of distance VI (based on the worse eye) from this programme was similar to one population-based study conducted in another district (Shatin) 20 years ago which reported a 41.3% prevalence of VI with VA worse than 6/18 in at least one eye [6]. It is worth noting that the sampling methods were different. The Shatin study was a population-based study that used cluster sampling while convenient sampling was used in our study. Although the sampling methods were different, both studies ended up with a similar demographic profile of subjects (mean age: ~ 70 years, female/male ratio: 6:4). It is worth noting that a pinhole measurement instead of a subjective refraction was used in the Shatin study. The prevalence of VI was reduced from 41.3% to 34.5% using pinhole VA, a 16.5% relative reduction in the VI. The pinhole reduces the influence of spherical aberration and diffractive effects of media opacities. An improvement in VA with a pinhole does not imply a refractive error [11]. The pinhole acuity may also be several letters worse than the subjective BCVA [12].

Among the 1227 subjects who were still visually impaired after refractive error correction, the common suspected ocular disorders were anterior eye disorders including cataracts, pterygium, corneal degenerations and dystrophies, and posterior eye disorders including age-related macular degeneration, myopic maculopathy, epiretinal membrane, diabetic retinopathy, glaucoma and macular hole. These cases were advised to seek medical attention through the usual channels consistent with local practice.

One limitation of this study was that no data was collected on individual socioeconomic status, knowledge about ageing and vision problems, or other health data. Consequently, we were not able to perform further data analysis to determine the factors associated with VI. Moreover, the scheme excluded applicants receiving CSSA and fringe benefits (such as civil servants). This could result in a selection bias and underestimation of the prevalence of VI if the excluded groups were associated with a higher risk of VI. Besides these limitations, other limitations had been mentioned in previously published studies. For example, in defining VI, only central visual acuity was used although it is accepted that visual field integrity is an important element of visual performance. This problem is often faced by people with glaucoma, whereby progressive visual field loss and constriction may not be associated with a concurrent drop in VA [13]. It is possible that some of our subjects could have good central vision but constricted visual fields. Near vision is also an important element in enabling activities of daily living, but near VI was not considered in this study. In the majority of the cases, near vision can also be improved with near optical correction if the distance vision is correctable and therefore avoiding near VI. However, a more detailed study will be helpful to investigate issues related to near vision for the elderly population. As activities of daily living become increasingly centred on small portable screens, near vision performance is becoming more important to the quality of life.

This is a large-scale community-based programme providing vision care to over 8000 elders. From the funder’s perspective, we estimated the direct cost of such a subsidised programme and associated immediate benefit in terms of avoiding distance VI by refractive error correction during the comprehensive vision examination. We estimated that it costs between HK$1921 and HK$3715 to prevent one distance VI case. This represents the short-term cost-effectiveness of such a subsidised programme versus no subsidy, and provides a quantifiable basis to consider whether or not to provide subsidised vision care for the elderly. Future research should look holistically at the societal level and the cost–benefit of minimizing VI in the ageing population. This future research could further consider the impact of VI on elders’ quality of life and comprehensive costs across a lifetime, such as the cost of health services and social care services, social welfare payment, and the cost of caregivers depending on the perspective. A previous study estimated that the global cost of establishing and operating educational and refractive care facilities to deal with VI resulting from uncorrected refractive error was far below the global loss in productivity associated with that VI [14]. Another study reported that a dilated eye evaluation was cost-effective when compared to no screening [15].

Elders can benefit from such a community-based programme which could potentially result in better daily physical function, quality of life and independence by avoiding VI. Recent systematic reviews and meta-analyses found that VI was significantly associated with cognitive impairment in cross-sectional and longitudinal studies [16, 17]. In a longitudinal cohort study, it was found that worse visual acuity at baseline was associated with greater declines in language and memory domains of cognitive function [18]. Although the causality between the VI and cognitive impairment remains to be investigated, the value of screening elders for visual impairment and optimizing their vision could potentially contribute to the prevention of cognitive decline.

## Conclusions

The prevalence of distance VI was relatively high among older people in HK. The main cause was uncorrected refractive errors. Having access to regular vision care and affordable prescription spectacles would reduce the prevalence of VI and associated risks.

## Data Availability

The dataset analyzed for during the current study is not publicly available as the service recipients’ data is protected by the Personal Data (Privacy) Ordinance and ethical approval on the use of the data was restricted to the approved research proposal only. Please contact the corresponding author if there are any queries regarding the dataset.

## Abbreviations

VI: Visual Impairment
VA: Visual Acuity
WHO: World Health Organization
HK: Hong Kong
KT: Kwai Tsing
PolyU: The Hong Kong Polytechnic University
ICHC: The Integrative Community Health Centre
CSSA: The Comprehensive Social Security Assistance
